# Supplementary material for: Human placental mesenchymal stem cells ameliorates premature ovarian insufficiency via modulating gut microbiota and suppressing the inflammation in rats
Source: PLoS One. 2025 Mar 5;20(3):e0313763. doi: 10.1371/journal.pone.0313763 (PMC11882084; doi:10.1371/journal.pone.0313763)
Supplement: S1 File — Datasets of each histogram. (DOCX) [file pone.0313763.s001.docx]

DATASETS OF EACH HISTOGRAM:

Figure 4

|  | CON | MED | MOD | PMSC |
| --- | --- | --- | --- | --- |
| FIG 4A | 58.8 | 36.4 | 44.5 | 32.9 |
|  | 66.8 | 38 | 50 | \| 75.2 \| \| --- \| |
|  | 57.6 | 32 | 40 | 53.8 |
|  | 61.07±5.001 | 35.47±3.107 | 44.83±5.008 | 60.63±12.62 |
|  | CON | MED | MOD | PMSC |
| FIG 4B | 0.459 | 0.4659 | 0.278 | 0.4279 |
|  | 0.5 | 0.5319 | 0.386 | 0.506 |
|  | 0.42 | 0.414 | 0.249 | 0.462 |
|  | 0.4597±0.04 | 0.4706±0.05909 | 0.4706±0.05909 | 0.4653±0.03915 |

| Figure 7 |  |
| --- | --- |

|  | CON | MED | MOD | PMSC |
| --- | --- | --- | --- | --- |
| FIG 7A | 627.5874793 | 605.5308231 | 736.5125685 | 670.766254 |
|  | 578.4198081 | 476.5632503 | 739.7738442 | 598.957164 |
|  | 594.0791196 | 659.5494335 | 697.3315 | 541.1742765 |
|  | 600±25.12 | 580.5±94.02 | 724.5±23.62 | 603.6±64.92 |
|  | CON | MED | MOD | PMSC |
| FIG 7B | 1.824796524 | 1.817266576 | 1.615783404 | 1.809871756 |
|  | 1.848197136 | 1.844212579 | 1.613830924 | 1.712531539 |
|  | 1.6813731 | 1.7048344 | 1.630111531 | 1.983442476 |
|  | 1.785±0.09032 | 1.789±0.07393 | 1.62±0.00889 | 1.835±0.1372 |
|  | CON | MED | MOD | PMSC |
| FIG 7C | 0.617548497 | 1.544575271 | 0.178296633 | 0.568774497 |
|  | 0.787604016 | 1.264545324 | 0.139409172 | 0.502579853 |
|  | 0.588547165 | 0.56441448 | 0.547097713 | 0.013876404 |
|  | 0.743591993 | 0.508856317 | 0.084985175 | 0.941524044 |
|  | 0.6843±0.09628 | 0.9706±0.5145 | 0.2374±0.21 | 0.5067±0.3811 |
|  | CON | MED | MOD | PMSC |
| FIG 7D | 16.01515647 | 15.96145092 | 15.346317 | 16.17852948 |
|  | 16.28932512 | 16.12369575 | 15.855168 | 15.39550983 |
|  | 15.69856407 | 15.20099487 | 15.90812143 | 16.06923808 |
|  | 16.06923808 | 16.28932512 | 16.12369575 | 15.80259063 |
|  | 16.02±0.2438 | 15.89±0.4809 | 15.81±0.3292 | 15.86±0.3485 |
|  | CON | MED | MOD | PMSC |
| FIG 7E | 21.24529546 | 26.10816892 | 6.73902271 | 11.24893933 |
|  | 32.59352641 | 15.53731039 | 3.083413578 | 14.03115813 |
|  | 17.6009143 | 18.21273938 | 5.996324036 | 12.30525891 |
|  | 23.81±7.819 | 19.95±5.496 | 5.273±1.932 | 12.53±1.404 |
|  | CON | MED | MOD | PMSC |
| FIG 7F | 5.0761937 | 5.150951168 | 4.927211492 | 3.868333013 |
|  | 4.084348133 | 4.374854357 | 4.156708325 | 3.832485872 |
|  | 4.411367408 | 3.2649692 | 3.868333013 | 4.964390453 |
|  | 3.582798917 | 5.0761937 | 4.048234628 | 3.547306928 |
|  | 4.289±0.6259 | 4.467±0.8741 | 4.25±0.4668 | 4.053±0.6243 |
| Figure 8 | | | | |
|  | CON | MED | MOD | PMSC |
| FIG 8A  PLASMA | 59.60561425 | 30.587452 | 89.177088 | 42.980448 |
|  | 56.48914425 | 45.320352 | 92.705893 | 43.85478825 |
|  | 57.73073425 | 61.179468 | 97.716192 | 47.98460625 |
|  | 57.94±1.569 | 45.7±15.3 | 93.2±4.291 | 44.94±2.673 |
|  | CON | MED | MOD | PMSC |
| FIG 8A  OVARY | 104.6464563 | 116.276517 | 155.2529843 | 141.9616083 |
|  | 103.9098743 | 106.1246183 | 146.8958943 | 143.189557 |
|  | 97.35560625 | 143.189557 | 148.138837 | 139.5169563 |
|  | 102±4.014 | 121.9±19.15 | 150.1±4.509 | 141.6±1.87 |
|  | CON | MED | MOD | PMSC |
| FIG 8B  PLASMA | 18.49406487 | 18.57624632 | 18.55924375 | 18.28999152 |
|  | 18.30161443 | 18.37729783 | 18.41896192 | 18.50993488 |
|  | 18.37729783 | 18.478478 | 18.478478 | 18.62895287 |
|  | 18.32570967 | 18.50993488 | 18.41896192 | 18.54252432 |
|  | 18.37±0.08563 | 18.49±0.08284 | 18.47±0.06644 | 18.49±0.1443 |
|  | CON | MED | MOD | PMSC |
| FIG 8B  OVARY | 980.8427049 | 1134.355004 | 1551.273319 | 1450.394066 |
|  | 1072.593668 | 1254.087563 | 1648.77103 | 1257.57061 |
|  | 1106.14194 | 1042.98713 | 1679.336137 | 1296.444745 |
|  | 1053±64.86 | 1144±105.9 | 1626±66.88 | 1335±102 |
|  | CON | MED | MOD | PMSC |
| FIG 8C  PLASMA | 36.17889842 | 36.07629488 | 34.23382802 | 36.80634602 |
|  | 36.490088 | 35.9742545 | 36.07629488 | 40.4951345 |
|  | 36.07629488 | 36.490088 | 34.0517105 | 35.27574032 |
|  | 36.25±0.2155 | 36.18±0.2732 | 34.79±1.12 | 37.53±2.683 |
|  | CON | MED | MOD | PMSC |
| FIG 8C  OVARY | 319.5599629 | 725.8895 | 489.3422509 | 853.7085555 |
|  | 307.0627467 | 394.4527416 | 525.3864245 | 947.9327076 |
|  | 312.7095099 | 393.1668305 | 603.1415045 | 774.6284053 |
|  | 313.1±6.258 | 504.5±191.7 | 539.3±58.16 | 858.8±86.76 |

|  |
| --- |

|  | CON | MED | MOD | PMSC |
| --- | --- | --- | --- | --- |
| FIG 8D  PLASMA | 4.39077864 | 3.907711 | 4.74608904 | 4.96986364 |
|  | 4.568014 | 3.86411064 | 4.61245404 | 4.346601 |
|  | 4.74608904 | 3.95136384 | 3.907711 | 4.25840316 |
|  | 4.568±0.1777 | 3.908±0.04363 | 4.422±0.4504 | 4.525±0.3878 |
|  | CON | MED | MOD | PMSC |
| FIG 8D  OVARY | \| 113.785308 \| \| --- \| | 188.9441225 | 178.6025 | 145.8229541 |
|  | 127.040178 | 196.9200941 | 212.4028453 | 125.4184205 |
|  | \| 114.650598 \| \| --- \| | 199.5377539 | 213.303108 | 144.2961643 |
|  | 118.5±7.416 | 195.1±5.518 | 201.4±19.78 | 138.5±11.37 |

|  |
| --- |

|  | CON | MED | MOD | PMSC |
| --- | --- | --- | --- | --- |
| FIG 8E  PLASMA | 242.1461273 | 223.0501378 | 258.5398784 | 243.0112262 |
|  | 230.0082583 | 225.6613246 | 235.2162554 | 203.8320739 |
|  | 172.1210153 | 210.8345851 | 307.1748235 | 214.3297874 |
|  | 401.1688958 | 226.5312158 | 320.6794414 | 193.2980407 |
|  | 261.4±98.09 | 221.5±7.275 | 280.4±40.24 | 213.6±21.39 |
|  | CON | MED | MOD | PMSC |
| FIG 8F  PLASMA | 0.1078214 | 0.1084724 | 0.1115321 | 0.1099046 |
|  | 0.1057382 | 0.1048268 | 0.1119227 | 0.1071053 |
|  | 0.1085375 | 0.1099697 | 0.1100999 | 0.1054127 |
|  | 0.1074±0.001454 | 0.1078±0.002645 | 0.1112±0.0009597 | 0.1075±0.002269 |

|  |
| --- |
|  |
