# Supplementary material for: Human placental mesenchymal stem cells ameliorates premature ovarian insufficiency via modulating gut microbiota and suppressing the inflammation in rats
Source: PLoS One. 2025 Mar 5;20(3):e0313763. doi: 10.1371/journal.pone.0313763 (PMC11882084; doi:10.1371/journal.pone.0313763)
Supplement: Attachment — Submitted filename: PONE-D-24-36820-review by FGhiamati.docx [file pone.0313763.s002.docx]

Thanks for your exciting piece of work. I have listed some general and technical issues. However,
Due to the low resolution of figures 9-13 and the lack of line numbers, I couldn’t properly review the manuscript.

**General Issue,**

1- Define all abbreviations in the abstract
2- Show the positive or negative correlation of the mentioned phyla in the abstract and not just say: “remarkable difference.”
3- Mention the concentration of collagenase A and DNase for placenta cell isolation
4- Why didn’t you use the shaker incubator for digestion?
5- Mention the yield of cell isolation and the reference of the method you used
6- Figures 9-13 are of low quality, and I am not able to review the section of this manuscript that comprises them.

**Technical issues**

1- How come you used one fluorochrome (PE) for different markers (CD45, CD73, CD90, and CD105) and FITC for HLA-DR, CD34, and CD14? So, the flow cytometry analysis (Fig. 2E) is not understandable on the first page of your results.
2- I can not understand the meaning of Control cells in this sentence (At the same time, control cells continued to be added to the mesenchymal stem cells-specific culture medium.)
3- In the result section entitled:” **MSCs intervention ameliorated the homeostasis of steroid hormones in rats with POI,”** it seems that in the PMCS group, the level of LH is significantly higher than in MOD, which is not reflected in the graph. Can you check?
4- According to Figure 8, the LPS graph, all groups have the same LPS. Please change the information in the results accordingly
5- Please include the effect of PMCS regarding the microbial changes among these lines at page 22:

“Analysis results showed that the MOD group was enriched in
Rikenella, Micrococcaceae, Bacteroidales, and Desulfovibrionaceae, while the CON group enriched in Lachnospiraceae and Clostridia (Fig. 9E). These findings provided important clues for us to understand further the effects of PMSCs on POI by modulating the intestinal microbiota”.

6- The information you provided for this title:” **The difference in intestinal flora in rats of each group at the phylum level,” has been repeated:” The difference in intestinal flora in rats of each group at the genus level.”** You may need to omit from the second part.

Final Note: Despite having trouble interpreting the results for Figures 9-13, I believe that the discussion of gut microbiota, inflammatory cytokines, and SCFA was comprehensive and informative. Therefore, I asked the corresponding editor to send me the high-resolution figures and a version of the manuscript with the line number to finish my review.
